# Acupuncture Decreases NF-*κ*B p65, miR-155, and miR-21 and Increases miR-146a Expression in Chronic Atrophic Gastritis Rats

**DOI:** 10.1155/2016/9404629

**Published:** 2016-05-18

**Authors:** Jialing Zhang, Kangbai Huang, Guoxin Zhong, Yong Huang, Suhe Li, Shanshan Qu, Jiping Zhang

**Affiliations:** ^1^School of Traditional Chinese Medicine, Southern Medical University, Guangzhou, Guangdong 510515, China; ^2^Clinical Medical College of Acupuncture, Moxibustion and Rehabilitation, Guangzhou University of Chinese Medicine, Guangzhou, Guangdong 510006, China

## Abstract

Acupuncture has been used to treat chronic atrophic gastritis (CAG) in traditional Chinese medicine (TCM) for centuries. In this study, we evaluated the effect of acupuncture at Zusanli (ST36), Zhongwan (CV12), and Pishu (BL20) acupoints on weight changes of rats, histological changes of gastric glands, and expressions changes of nuclear factor-kappa B (NF-*κ*B) p65, microRNA- (miR-) 155, miR-21, and miR-146a in CAG rats induced by N-methyl-N′-nitro-N-nitrosoguanidine (MNNG) combined with irregular diet. Consequently, we found that acupuncture treatment elevated body weight of rats significantly when compared to the model group. By observing histological changes, we found that the acupuncture group showed better improvement of gastric mucosa injury than the model group. Our results also demonstrated upregulation of NF-*κ*B p65, miR-155, and miR-21 in gastric tissue of CAG rats and a positive correlation between miR-155 and miR-21. Relatively, expression of miR-146a was downregulated and negative correlation relationships between miR-146a and miR-155/miR-21 in CAG rats were observed. Additionally, expressions of NF-*κ*B p65, miR-155, and miR-21 were downregulated and miR-146a was upregulated after acupuncture treatment. Taken together, our data imply that acupuncture can downregulate NF-*κ*B p65, miR-155, and miR-21 and upregulate miR-146a expression in CAG rats. NF-*κ*B p65, miR-155, miR-21, and miR-146a may play important roles in therapeutic effect of acupuncture in treating CAG.

## 1. Introduction

Chronic atrophic gastritis (CAG), characterized by loss of normal gastric glandular structures and accompanied by intestinal metaplasia, is well known as a significant premalignant lesion of gastric cancer [1–3]. Recent researches have provided evidence of a sustained inflammatory reaction in gastritis with activation of immune response and involvement of inflammatory pathway [4–6]. With deepening researches on pathogenesis, treatments for CAG have attracted growing attention. However, except pharmacological agents such as* Helicobacter pylori* eradication, acid suppression, and nonsteroidal anti-inflammatory drug treatment, there is no more available effective treatment for CAG currently [3, 7, 8].

Acupuncture, originated in ancient China, is one of the most commonly used complementary medicine modalities in the world [9, 10]. A lot of researches have demonstrated the efficacy of acupuncture in treating gastric diseases [7, 11–13], especially CAG. Additionally, Zusanli (ST36), Zhongwan (CV12), and Pishu (BL20) acupoints were commonly used to treat CAG in China [7, 14, 15], which were included in clinical practice guideline of traditional Chinese medicine (TCM) for chronic gastritis [16]. Recently, it has been proposed that acupuncture could alleviate inflammatory responses through different inflammatory pathways in treating gastrointestinal lesion [17–19]. However, modulation effect of acupuncture in treating CAG remained inconclusive.

MicroRNAs (miRNAs) are a class of small noncoding RNAs [20], which have been discovered as crucial regulators in gastric carcinogenesis through posttranscriptional regulation [21]. Recently, increasing researches have suggested involvement of miRNAs in different processes of gastric carcinogenesis [21, 22], especially miR-155, miR-21, and miR-146a [22–24]. In addition, accumulating evidences have demonstrated the relationship between gastritis and the abovementioned miRNAs [22, 24–26]. Petrocca et al. showed that chronic gastritis was associated with the alteration of miR-155 [25], which was known to play a major role in regulation of immune response [27] and promote tumor progression [28]. Link et al. observed a gradual increase trend of miR-155 and miR-21 expression in preneoplastic gastric mucosa [22], including CAG stage, indicating that miR-155 and miR-21 were essential to persistent inflammation of gastric mucosa. Furthermore, Liu et al. demonstrated that overexpression of miR-146a in chronic gastritis could significantly decrease activity of nuclear factor-kappa B (NF-*κ*B) pathway [24], a well-known signaling pathway of inflammation response, suggesting that miR-146a might play a crucial role in a negative feedback loop to modulate gastric mucosa inflammation. In conclusion, previous researches have indicated that miR-155, miR-21, and miR-146a may function as inflammation regulators in CAG.

In this study, we evaluated the therapeutic effect of acupuncture on body weight changes and gastric histological structures and investigated expressions of NF-*κ*B p65, miR-155, miR-21, and miR-146a in CAG, thus to explore the therapeutic effect of acupuncture in treating CAG.

## 2. Materials and Methods

### 2.1. Animals

A total of 60 Sprague-Dawley (SD) rats, half male and half female, 8 weeks old, 180~220 g in weight, provided by the Medical Experimental Animal Center of Guangdong Province (permit number: SCXK (Yue) 2008-0002) were used. Rearing conditions were a laminar flow, specific pathogen-free (SPF) atmosphere, room temperature (20 ± 1)°C, relative humidity 50~60%, automatic ventilation 8~15 times per hour, and 12 h light/dark cycle. The study was carried out adhering to the guidelines provided by the National Institutes of Health for the Care and Use of Laboratory Animals and all efforts were made to minimize suffering of animals.

### 2.2. CAG Model Preparation [15, 29]

After 1 week of adaptive feeding, the study was begun when the rats behaved normally. A total of 60 rats were randomly divided into 2 groups for control (*n* = 20) and N-methyl-N′-nitro-N-nitrosoguanidine (MNNG) treatment (*n* = 40) by using completely randomized method based on SPSS software (Version 20.0, SPSS Inc., USA). Rats in the MNNG treatment group were induced by MNNG (100 *μ*g/L; Tokyo Kasei Kogyo Co., Ltd.) combined with irregular diet for 12 weeks. MNNG was dissolved in distilled water at a concentration of 1 g/L and kept in a cool (4°C) and dark place. Just before use, the stock solution was diluted to 100 *μ*g/L with distilled water. Rats in the model group were given MNNG solution from a bottle which was covered with aluminum foil to prevent photolysis of MNNG and used as only water source. The solution was replenished every day. In the meantime, rats in the MNNG treatment group were provided with irregular diet (one day of sufficient feeding and one day of fasting, alternating between the two). Rats in the control group were fed normally and given sterile water* ad libitum*. At week 12, from each group 2 rats were sacrificed to verify whether CAG models were successfully prepared by observing histological changes of gastric structures. After successful modeling (Figure S1 (in Supplementary Material available online at http://dx.doi.org/10.1155/2016/9404629) showed pathologic changes at various time points), rats in the MNNG treatment group were randomly divided into the model group (*n* = 18) and the acupuncture group (*n* = 20). Starting from week 12, all rats in each group were fed normally and given sterile water* ad libitum*. Rats were weighed weekly and their body weight changes were recorded.

### 2.3. Groups and Treatment

**Figure 1:**
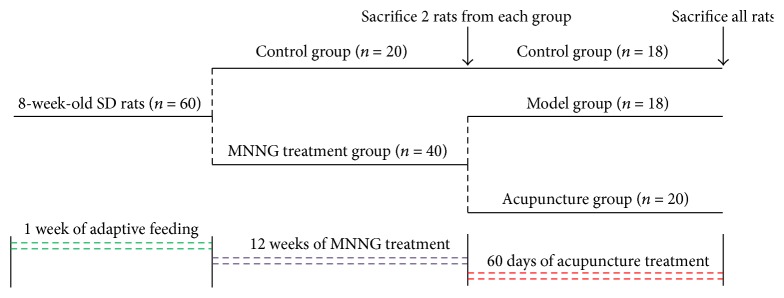
Flow diagram of the study.

Acupuncture group: starting from week 12, rats in the acupuncture group were given acupuncture treatment daily for 60 consecutive days. Zusanli (ST36, 5 mm lateral to the anterior tubercle of tibia [30], bilateral), Zhongwan (CV12, 9/14 above the pubic crest of the distance measured between the top of the xiphoid process and the pubic crest [30]), and Pishu (BL20, lateral to the lower border of the 11th thoracic vertebra in the back [31], bilateral) acupoints were selected. Rats were lightly immobilized, and the acupuncture needles (Suzhou Medical Appliance Factory; 0.25 mm × 15 mm) were inserted to a depth of 3 mm at the acupoints and retained for 15 min. In the control group, no treatment was performed. In the model group, the same grasping and fixing as the acupuncture group was performed. No rat died in the treatment stage. After acupuncture treatment was finished, all rats in each group were anesthetized and their stomachs were quickly removed.  Figure 1 is a flow diagram of the study.

### 2.4. Histopathological Observation

Gastric tissue samples from each group were collected and fixed in 10% formalin (Invitrogen). Then the samples were embedded in paraffin after tissue was processed. This was followed by sectioning (5 *μ*m thickness) and staining with hematoxylin and eosin (H&E) dye. The sections were observed and analyzed using light microscopy and photographed. Histological score was assessed by the diagnostic criteria of gastritis in Houston in 1996 [32, 33]. Inflammatory score was calculated in 10 microscopic fields of each section and ranked in a 4-score system [34] (0 = normal; 1 = mild, few inflammatory cells' infiltration in pit or basal region of gastric glands; 2 = moderate amount of inflammatory cells infiltration, which localized within two-thirds of gastric glands; 3 = marked, large number of inflammatory cells' infiltration into whole gastric glands). Similarly, atrophy score of gastric glands was calculated in 10 microscopic fields of each section and ranked in a 4-score system (0 = normal; 1 = mild, atrophy less than one-third of gastric glands; 2 = moderate, atrophy localized within two-thirds of gastric glands; 3 = marked, atrophy more than two-thirds of gastric glands). Histological score was composed of inflammatory score and atrophy score. Besides, morphology and structure of chief cells were also observed by transmission electromicroscope.

### 2.5. Immunohistochemistry

Expression of NF-*κ*B p65 in gastric tissue was detected with streptavidin-biotin-peroxidase complex (SABC) immunostain kit (Abcam Co., USA) according to the manufacturer's instructions. Paraffin embedded tissue sections were prepared at 2 *μ*m thickness. After deparaffinization, antigen retrieval was undertaken by high pressure in a buffer composed of sodium citrate (0.01 mol/L). Endogenous peroxidase was cleared with 3% hydrogen peroxide. After blocking with 10% normal goat serum, the sections were incubated with monoclonal antibodies against NF-*κ*B p65 of rabbit anti-rat (1 : 150 dilution, Abcam Co., USA) overnight at 4°C. Afterward, a streptavidin-peroxidase assay kit (Rui Shu Biological Technology Co., Ltd.) was used to develop antibody signal. DAB (Rui Shu Biological Technology Co., Ltd.) was used for staining and hematoxylin for counterstaining. PBS was used as the first antibody for negative control. Images were acquired with microscope and analyzed by Image-Pro Plus 6.0 image system. Under the microscope magnifying at 400 times, 10 random sights of each section were selected to conduct the semiquantitative analysis of the average optical density (OD).

### 2.6. Real-Time PCR

Total RNA was extracted from frozen tissues using TRIzol reagent (Invitrogen). The RNA concentration was quantified by NanoDrop® ND-1000. Extracted RNA was reverse transcribed into complementary DNA using MMLV reverse transcriptase (Epicentre) and RT primers (Invitrogen). RT-PCR was carried out using TaqMan probes (Applied Biosystems) according to the manufacturer's instructions, with Gene Amp PCR System 9700 (Applied Biosystems). All reactions were run in triplicate, and average threshold cycle number (Ct) data of each miRNA was recorded. miRNAs expression in cells was normalized to U6 small noncoding RNA.

The ΔCt method and 2^−ΔΔCt^ method were used for analysis. The ΔCt value was the difference between the Ct value of the specific miRNA and the Ct value of U6, ΔCt = Ct (miRNA) − Ct (U6). ΔΔCt = ΔCt (sample) − ΔCt (reference). 2^−ΔCt^ represented miRNAs expression of each sample. 2^−ΔΔCt^ represented the expression relative quotient (RQ) of target RNA to control RNA.

### 2.7. Statistical Analysis

The results were expressed as mean ± SD. One-way analysis of variance (ANOVA) followed by Bonferroni post hoc tests was used to compare contents of miRNAs in different groups; repeated measures analysis of variance was used to compare body weights among groups, using SPSS software (Version 20.0, SPSS Inc., USA). Pearson's test was applied to analyze correlations of RQ values of miRNAs. *P* < 0.05 was considered to be statistically significant.

## 3. Results

### 3.1. Body Weight Changes

Table 1 showed body weight changes in different groups. Body weights in the model group and the acupuncture group were significantly lower than the control group at week 8, week 12, and 60 days after treatment (*P* < 0.001). However, after 60 days of acupuncture treatment, body weights in the acupuncture group were significantly higher than that in the model group (*P* < 0.001).

### 3.2. Histopathological Evaluation

**Figure 2:**
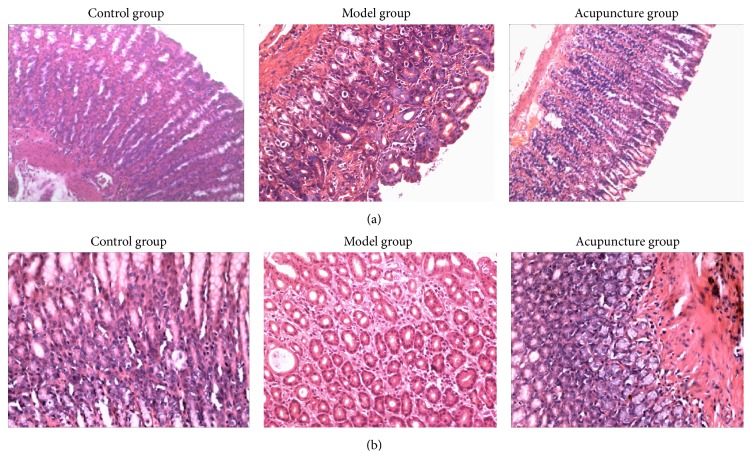
Histological evaluation of gastric glands in rats. (a) H&E (×100) staining of gastric glands. Rats in the control group showed complete glandular structure (left diagram). Rats in the model group showed irregular arrangement and reduction of gastric glands with atrophic gastritis (middle). Rats in the acupuncture group showed regular arrangement and increase of gastric glands after acupuncture treatment (right diagram). (b) H&E (×200) staining of gastric glands. Rats in the model group showed cystic dilation and neutrophils and lymphocytes infiltrated into gastric glands with atrophic gastritis (middle). Rats in the acupuncture group showed an increased number of gastric glands and inflammatory cells were reduced (right diagram).

Histological observation of gastric lesion induced by MNNG combined with irregular diet in the model group showed cystic dilation, irregular arrangement, reduction, and inflammatory cells infiltration in gastric glands. On the other hand, the acupuncture group showed relatively better protection of gastric mucosa as seen by regular arrangement and increased number of gastric glands and significant reduction in inflammatory cells infiltration. Relatively, gastric mucosa tissue in the control group showed intact appearance of gastric glands when compared with the model group, as shown in Figure 2.

It could be observed that inflammatory score, atrophy score, and histological score in the model group were significantly elevated when compared to the control group (*P* < 0.001). These data indicated that the experimental CAG in rats was successfully established. After acupuncture treatment, scores in the acupuncture group were significantly decreased when compared to the model group (*P* < 0.001). It could be indicated that acupuncture treatment had significant effects on improving structure of gastric glands, as shown in Table 2.

**Figure 3:**
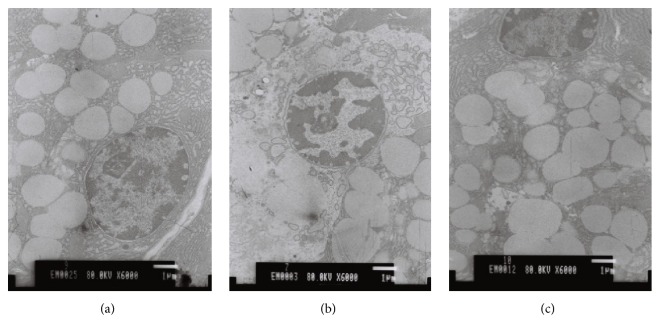
Histological evaluation of chief cells by TEM (×6000). (a) Control group. (b) Model group. TEM observation showed dilation of endoplasmic reticulum and reduction of Golgi complex and zymogen granules in chief cells in rats with atrophic gastritis. (c) Acupuncture group. TEM observation showed increase of endoplasmic reticulum, Golgi complex, and zymogen granules in chief cells in rats that received acupuncture treatment.

We also evaluated the morphology and structure of chief cells by transmission electromicroscope (Figure 3). In the model group dilation of endoplasmic reticulum and reduction of Golgi complex and zymogen granules in gastric chief cells in rats with CAG could be observed. On the contrary, after acupuncture treatment, increase of endoplasmic reticulum, Golgi complex, and zymogen granules in gastric chief cells was observed in rats of the model group.

### 3.3. Expression of NF-*κ*B p65

**Figure 4:**
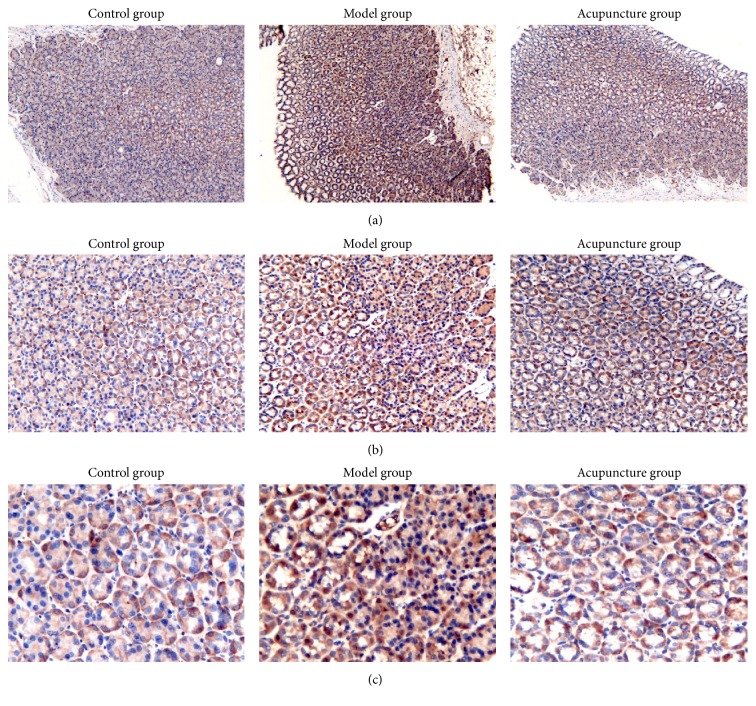
Immunohistochemistry stains of NF-*κ*B p65 in gastric glands. (a) ×100. (b) ×200. (c) ×400.

Figure 4 and Table 3 showed expression of NF-*κ*B p65 detected by immunohistochemical staining. Expression of NF-*κ*B p65 was significantly higher in the model group than in the control group. Moreover, expression of NF-*κ*B p65 was significantly decreased in the acupuncture group compared to that in the model group, and there was no significant difference between the acupuncture group and the control group.

### 3.4. 2^−ΔCt^ Values of miR-155, miR-21, and miR-146a

Table 4 (and Figure S2) showed 2^−ΔCt^ values of miRNAs obtained from different groups. Expression levels of miR-155 and miR-21 were upregulated significantly in the model group compared to those in the control group and downregulated significantly in the acupuncture group compared to those in the model group, and there was no significance between the acupuncture and control group. Relatively, expression level of miR-146a was downregulated significantly in the model group compared to that in the control group and upregulated significantly in the acupuncture group compared to that in the model group, and there was no significance between the acupuncture and control group.

### 3.5. Correlations among miR-155, miR-21, and miR-146a

Fold change (RQ = 2^−ΔΔCt^) values of miR-155, miR-21, and miR-146a were shown in Table 5. Pearson's test results indicated that there were a positive correlation relationship between miR-155 and miR-21 and negative correlation relationships between miR-146a and miR-155/miR-21, respectively.

## 4. Discussions

Previous researches have demonstrated that acupuncture has significant clinical efficacy in CAG patients, with improving pathological changes and clinical symptoms [7, 14, 35]. Similarly, by observing body weight changes, we found that acupuncture therapy could help in gaining weight in CAG rats. We also found that gastric mucosa injuries of CAG rats that received acupuncture therapy were significantly improved compared to those in the model group. Furthermore, we found upregulation of NF-*κ*B p65 in the model group and downregulation in the acupuncture group indicated that acupuncture could alleviate the inflammation reaction in gastric mucosa. These provide experimental evidences for effectiveness of acupuncture in treatment of CAG.

Previous studies have suggested the involvement of miRNAs in CAG; we further proved that expressions of miR-155 and miR-21 were upregulated and miR-146a was downregulated in gastric tissues of CAG rats. Previous studies have found that acupuncture elicited remarkable miRNAs profiling changes in rats [36, 37]; our finding further demonstrated that acupuncture could regulate miRNAs expressions in CAG rats. To be specific, expressions of miR-155 and miR-21 were downregulated and miR-146a was upregulated after acupuncture treatment. Additionally, our results indicated that there was a positive correlation relationship between miR-155 and miR-21, indicating synergistic effects of these two miRNAs, as what has been reported before [38]. And there were negative correlation relationships between miR-146a and miR-155/miR-21, respectively, indicating antagonism effects of them on CAG. The abovementioned findings suggested that miR-155, miR-21, and miR-146a were involved in the pathogenesis of CAG and might play an important role in modulation effect of acupuncture in treatment of CAG.

It is well established that miRNAs may serve as functional inflammation regulators in many diseases [39]. Previous researches have shown that miR-155, miR-21, and miR-146a were associated with gastritis [22, 24–26], indicating that miR-155, miR-21, and miR-146a may function as inflammation regulators in CAG. Moreover, miR-155 has been suggested as a crucial effector of immune response in gastric epithelial cell lines and gastric mucosal tissues, which upregulated by activating NF-*κ*B and activator protein-1 pathways [40]. And overexpression of miR-155 could negatively regulate release of proinflammatory cytokines, leading to chronic infection [24]. Similarly, miR-21 was also significantly overexpressed in CAG antrum mucosa [22], which upregulated by activating NF-*κ*B and cyclooxygenase-2/prostaglandin signaling [41, 42]. And knockdown of NF-*κ*B by a specific inhibitor could markedly suppress expression of miR-21 [42], which could slow down the process of gastric cancer. Another important miRNA associated with gastritis is miR-146a. Previous researches have demonstrated that downregulation of miR-146a expression could trigger inflammation response via NF-*κ*B-dependent immunity signaling by increasing thymic stromal lymphopoietin pathway (TSLP) level [43]. These results have suggested that miR-155, miR-21, and miR-146a are potential targets of NF-*κ*B, which are involved in an important signaling pathway in CAG [44], and efficacy of acupuncture in treatment of CAG may take effect by modifying expressions of miR-155, miR-21, and miR-146a via NF-*κ*B pathway, thus to alleviate inflammation reaction of gastric mucosa. Therefore, our findings implied that acupuncture may act through transcription factors and subsequent epigenetic changes, such as NF-*κ*B-miR-155/miR-21/miR-146a signaling. Additionally, significant improvements of histological changes in CAG rats after altering NF-*κ*B/miR-155/miR-21/miR-146a expression levels are powerful evidence to validate therapeutic roles of NF-*κ*B-miR-155/miR-21/miR-146a signaling in response to acupuncture treatment. What is more, downstream targets of miR-155/miR-21/miR-146a including I-kappa B kinase epsilon, Fas-associated death domain protein [40], and TSLP [43] have been reported. However, there is no definite conclusion on downstream targets of NF-*κ*B-miR-155/miR-21/miR-146a signaling. In conclusion, we proposed that acupuncture may exert its therapeutic effects via NF-*κ*B-miR-155/miR-21/miR-146a signaling, including (1) changes of transcription factors (such as NF-*κ*B); (2) changes of miRNAs (miR-155/miR-21/miR-146a); (3) changes of downstream targets (such as TSLP, remaining inconclusive). These changes resulted in remarkable therapeutic effects of acupuncture in CAG rats (as shown in Figure S3).

Possible limitations of the study include the fact that exact function of miR-155/miR-21/miR-146a, interaction between NF-*κ*B, miR-155, miR-21, and miR-146a, and existence of NF-*κ*B-miR-155/miR-21/miR-146a signaling and its definite downstream targets in response to acupuncture therapy remain inconclusive, which require further researches in the future work.

## Supplementary Material

Here are histology images in model group at various time points. As shown in Figure S1A, there is no obvious glandular atrophy at the end of week 8th. Relatively, at the end of week 12th (Figure S1B), histological observation showed that neutrophils and lymphocytes infiltration, cystic dilation, irregular arrangement and reduction of gastric glands were observed in rats with CAG, which met the diagnostic criteria. After 2 months withdraw of MNNG (Figure S1C), histological observation indicated that the characteristic changes (inflammatory infiltration, cystic dilation and reduction of gastric glands) of CAG still remained in model group. Consequently, it could be concluded that the CAG rat models induced by MNNG combined with irregular diet were stable. Figure S2 showed 2^‒ΔCt^ values of miRNAs obtained from different groups. Expression levels of miR-155 and miR-21 were up-regulated significantly in model group than in control group and down-regulated significantly in acupuncture group than in model group, and there was no significance between acupuncture and control group. Relatively, expression level of miR-146a was down-regulated significantly in model group than in control group and up-regulated significantly in acupuncture group than in model group, and there was no significance between acupuncture and control group. Figure S3 showed that acupuncture may exerts its therapeutic effects via NF-*κ*B-miR-155/miR-21/miR-146a signaling, which including (1) changes of transcription factors (such as NF-*κ*B, which evoked by *H. pylori* infection, physical damage or chemical damage); (2) changes of miRNAs (miR-155/miR-21/miR-146a); (3) changes of downstream targets (such as TSLP, remain inconclusive).

## Figures and Tables

**Table 1:** Body weight in different groups at various time points ($\overset{-}{X}\pm S$).

Group	*N*	Before MNNG treatment	Week 8	Week 12	60 days later
Control group	18	201.45 ± 8.95	276.62 ± 10.21	340.33 ± 11.54	413.90 ± 10.69
Model group	18	200.98 ± 9.46	263.09 ± 9.09^*∗*^	301.81 ± 10.88^*∗*^	339.15 ± 13.79^*∗*^
Acupuncture group	20	201.50 ± 9.05	261.75 ± 8.61^*∗*^	300.83 ± 10.47^*∗*^	374.21 ± 12.88^*∗*△^

Note: at week 8/week 12/60 days later, compared with the control group, ^*∗*^ *P* < 0.001. 60 days later, compared with the model group, ^△^ *P* < 0.001. Repeated measures analysis of variance was used.

**Table 2:** Histological scores of gastric glands in different groups ($\overset{-}{X}\pm S$).

Group	*N*	Inflammatory score	Atrophy score	Histological score
Control group	9	1.04 ± 0.23	0.21 ± 0.11	1.26 ± 0.27
Model group	9	2.56 ± 0.42^*∗*^	2.11 ± 0.19^*∗*^	4.70 ± 0.60^*∗*^
Acupuncture group	9	1.30 ± 0.22^△^	0.70 ± 0.22^*∗*△^	2.00 ± 0.25^*∗*△^

Note: compared with the control group, ^*∗*^ *P* < 0.001. Compared with the model group, ^△^ *P* < 0.001. ANOVA (2-tailed) was used.

**Table 3:** Average optical density of NF-*κ*B p65 in different groups ($\overset{-}{X}\pm S$).

Group	*N*	NF-*κ*B p65 (OD·*μ*m^−2^)
Control group	9	0.180 ± 0.100
Model group	9	0.290 ± 0.097^*∗*^
Acupuncture group	9	0.217 ± 0.044^△^

Note: compared with the control group, ^*∗*^ *P* < 0.01. Compared with the model group, ^△^ *P* < 0.01. ANOVA (2-tailed) was used.

**Table 4:** 2^−ΔCt^ values of miR-155, miR-21, and miR-146a.

Group	*N*	miR-155	miR-21	miR-146a
Control group	9	0.034 ± 0.008	10.103 ± 2.961	5.962 ± 1.063
Model group	9	0.056 ± 0.011^*∗*^	25.905 ± 3.702^*∗*^	1.065 ± 0.336^*∗*^
Acupuncture group	10	0.042 ± 0.008^△^	14.049 ± 3.601^△△^	5.302 ± 0.978^△△^

Note: compared with the control group, ^*∗*^ *P* < 0.001. Compared with the model group, ^△^ *P* < 0.05 and ^△△^ *P* < 0.001. ANOVA (2-tailed) was used.

**Table 5:** Fold changes of the expression of miR-155, miR-21, and miR-146a.

miRNAs	*r*	*P* value
miR-155 and miR-21	0.722	<0.001
miR-155 and miR-146a	−0.616	<0.05
miR-21 and miR-146a	−0.768	<0.001

Note: Pearson's test (2-tailed) was used.
