# Supplementary material for: Acupuncture Decreases NF-κB p65, miR-155, and miR-21 and Increases miR-146a Expression in Chronic Atrophic Gastritis Rats
Source: Evid Based Complement Alternat Med. 2016 May 18;2016:9404629. doi: 10.1155/2016/9404629 (PMC4887647; doi:10.1155/2016/9404629)
Supplement: Supplementary file 1 — Here are histology images in model group at various time points. As shown in Figure S1A, there is no obvious glandular atrophy at the end of week 8th. Relatively, at the end of week 12th (Figure S1B), histological observation showed that neutrophils and lymphocytes infiltration, cystic dilation, irregular arrangement and reduction of gastric glands were observed in rats with CAG, which met the diagnostic criteria. After 2 months withdraw of MNNG (Figure S1C), histological observation indicated that the characteristic changes (inflammatory infiltration, cystic dilation and reduction of gastric glands) of CAG still remained in model group. Consequently, it could be concluded that the CAG rat models induced by MNNG combined with irregular diet were stable. Figure S2 showed 2‒ΔCt values of miRNAs obtained from different groups. Expression levels of miR-155 and miR-21 were up-regulated significantly in model group than in control group and down-regulated significantly in acupuncture group than in model group, and there was no significance between acupuncture and control group. Relatively, expression level of miR-146a was down-regulated significantly in model group than in control group and up-regulated significantly in acupuncture group than in model group, and there was no significance between acupuncture and control group. Figure S3 showed that acupuncture may exerts its therapeutic effects via NF-κB-miR-155/miR-21/miR-146a signaling, which including (1) changes of transcription factors (such as NF-κB, which evoked by H. pylori infection, physical damage or chemical damage); (2) changes of miRNAs (miR-155/miR-21/miR-146a); (3) changes of downstream targets (such as TSLP, remain inconclusive). [file 9404629.f1.docx]

**Supplementary Information**

**1.** Histology images with H&E staining in model group at various time points (**Fig S1**).

| 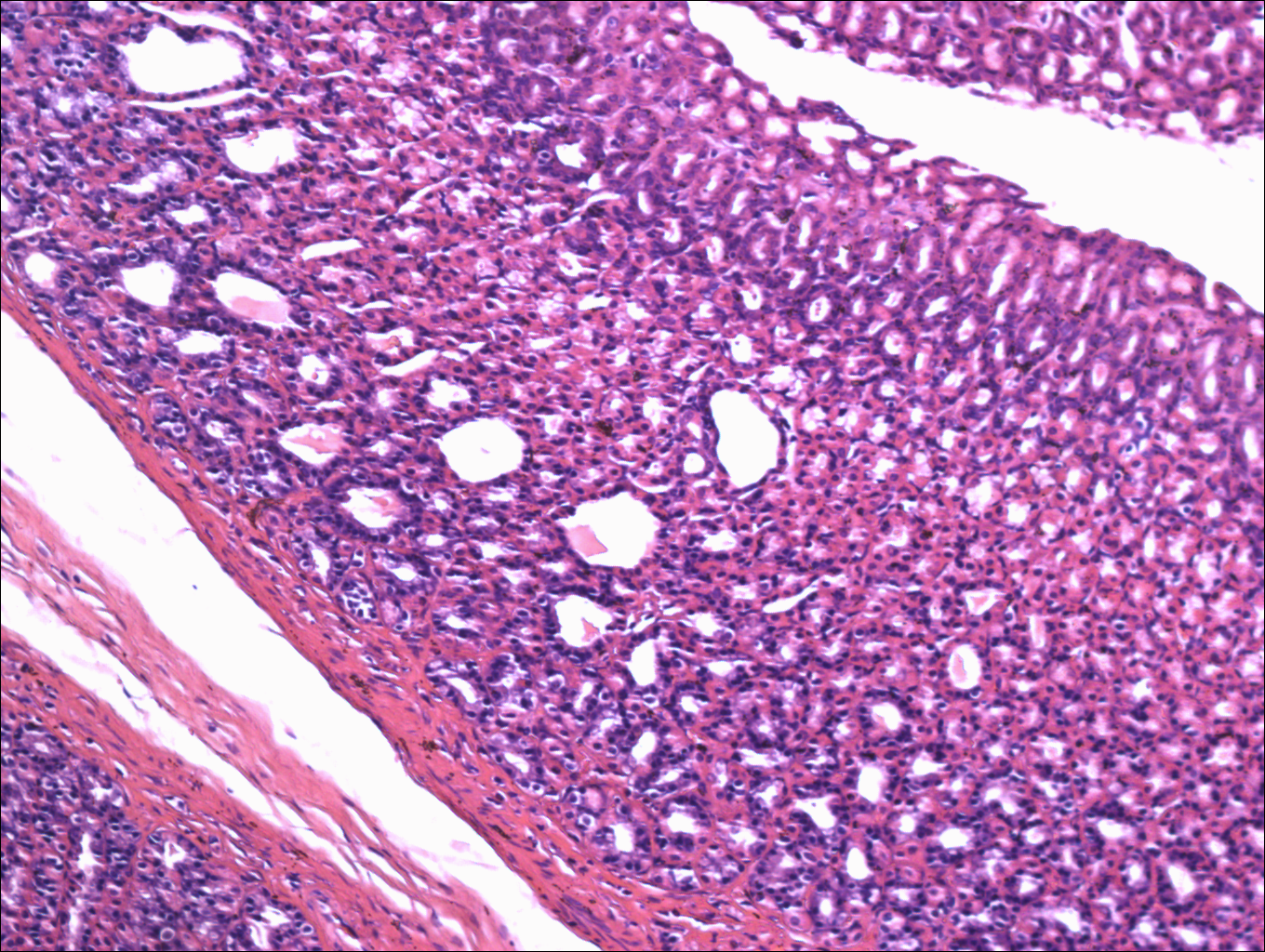 | 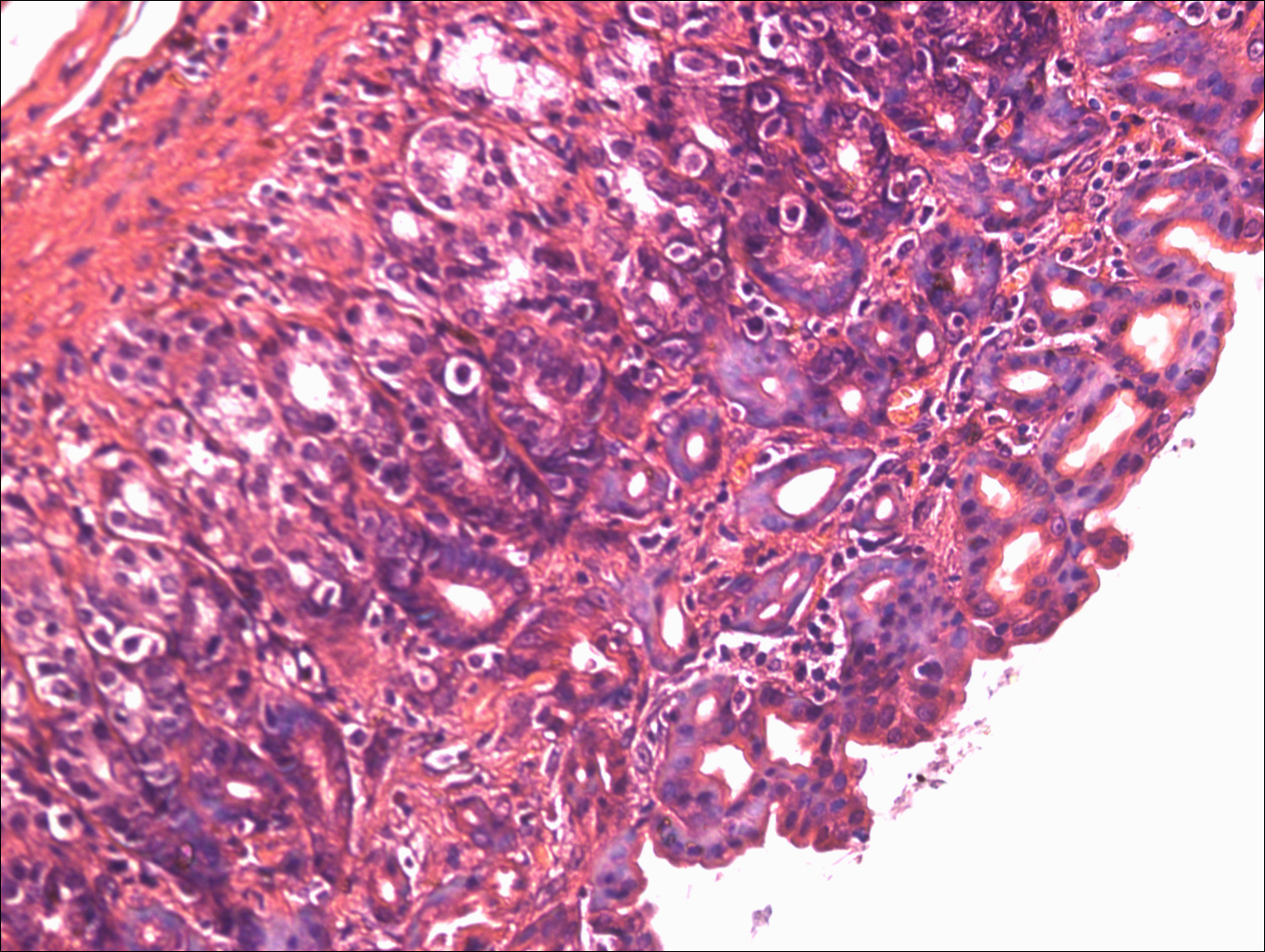 | 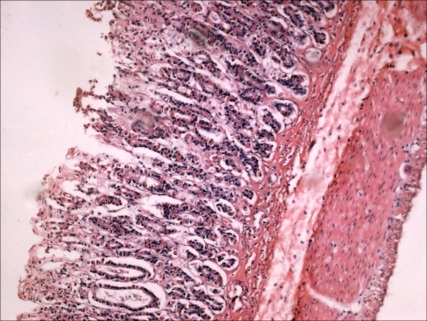 |
| --- | --- | --- |
| (A) | (B) | (C) |

**Fig S1. Histology images in model group at various time points.** (A) At the end of week 8^th^, histological observation showed cystic dilation and irregular arrangement of gastric glands without obvious atrophy. (B) At the end of week 12^th^, histological observation showed inflammatory infiltration, cystic dilation, irregular arrangement and reduction of gastric glands in rats with CAG. (C) After 2 months withdraw of MNNG, inflammatory infiltration, cystic dilation and reduction of gastric glands still remained in model group. H&E staining, ×100.

**2.** 2^-ΔCt^ values of miR-155, miR-21 and miR-146a from different groups (**Fig S2**).


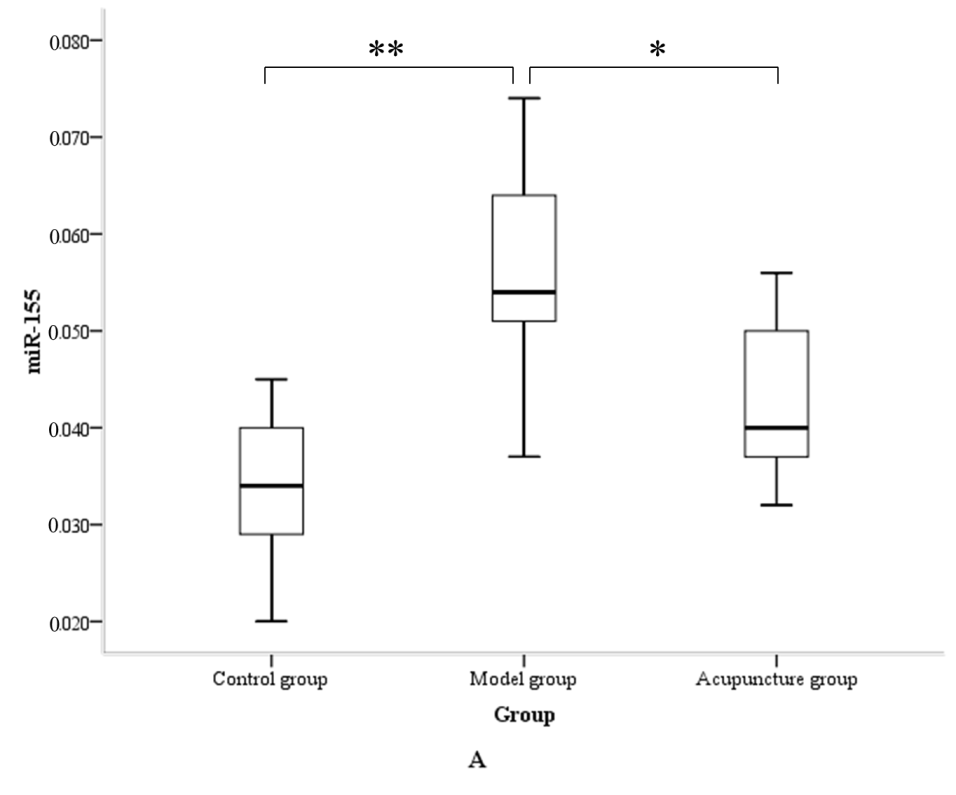


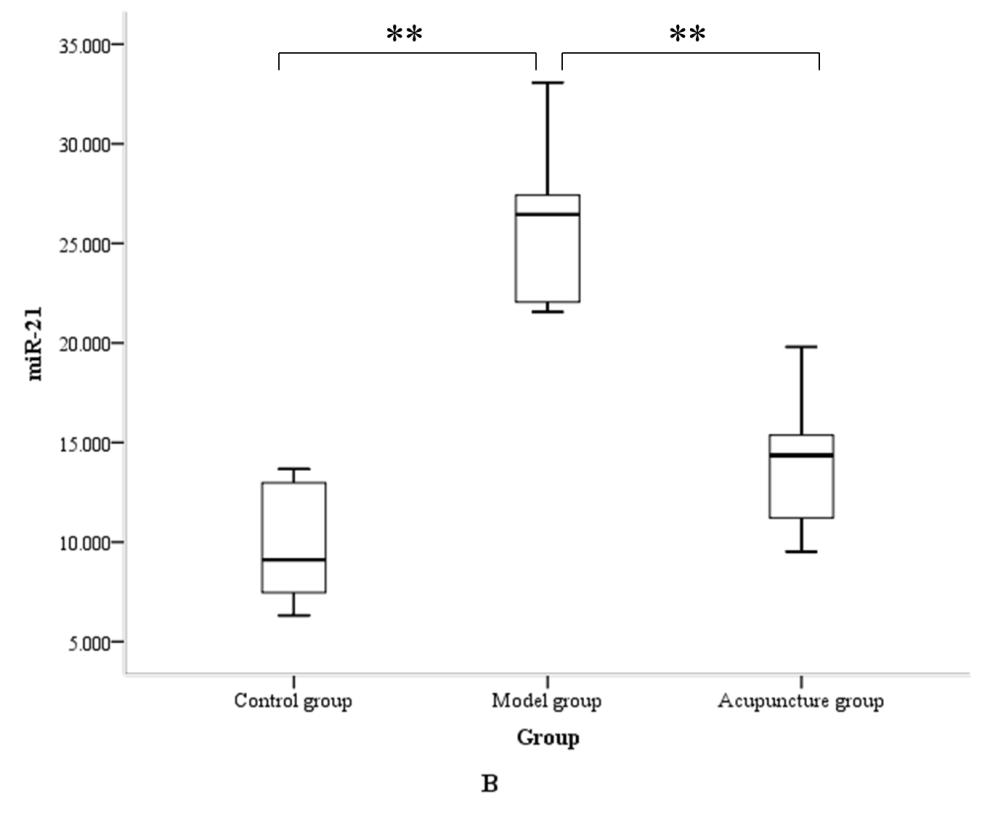


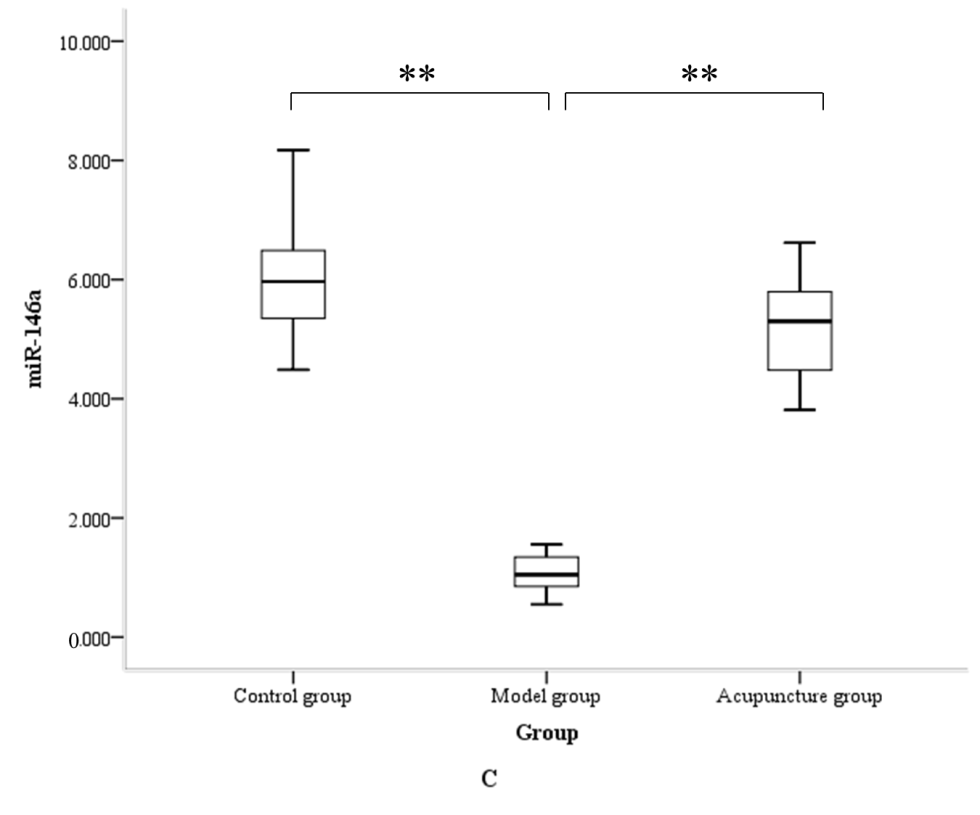


**Fig S2.** **2^-ΔCt^ values of miR-155, miR-21 and miR-146a from different groups.** (A), (B) and (C) represented 2-^ΔCt^ values of miR-155, miR-21 and miR-146a respectively. **P*<0.05, ***P*<0.001.

**3.** Proposed mechanisms of therapeutic effect of acupuncture through NF-κB-miR-155/-21/-146a signaling (**Fig S3**).

**
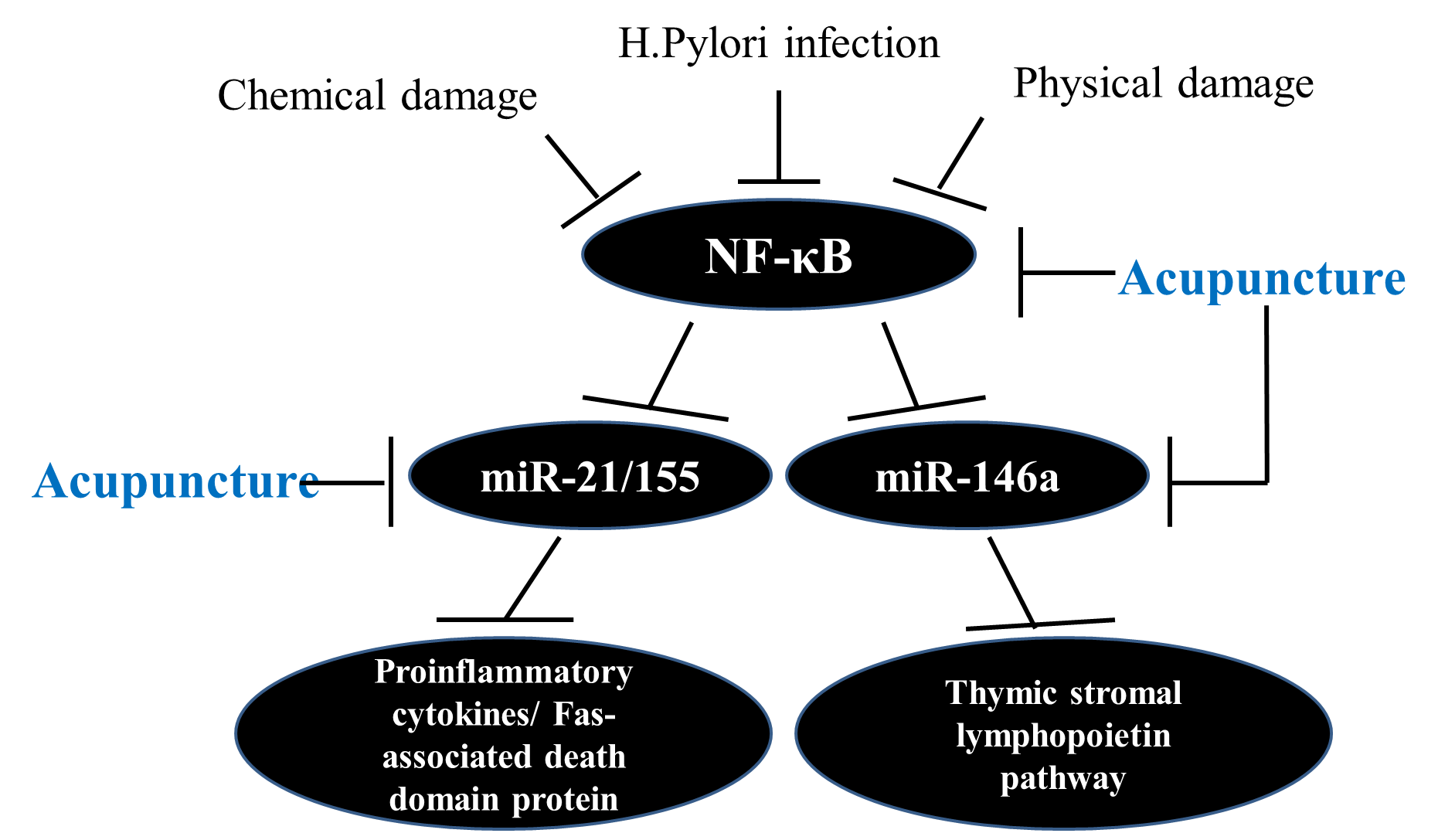
**

**Fig S3.** **Proposed mechanisms of therapeutic effect of acupuncture through NF-κB-miR-155/-21/-146a signaling.**
